# Expression of verotoxin-1 receptor Gb3 in breast cancer tissue and verotoxin-1 signal transduction to apoptosis

**DOI:** 10.1186/1471-2407-9-67

**Published:** 2009-02-26

**Authors:** David Johansson, Eldina Kosovac, Jasmin Moharer, Ingrid Ljuslinder, Thomas Brännström, Anders Johansson, Parviz Behnam-Motlagh

**Affiliations:** 1 Department of Medical Biosciences, Clinical Chemistry, Umeå University, SE-901 85 Umeå, Sweden; 2 Department of Radiation Sciences, Oncology, Umeå University, SE-901 85 Umeå, Sweden; 3 Department of Medical Biosciences, Pathology, Umeå University, SE-901 85 Umeå, Sweden; 4 Department of Odontology, Periodontology, Umeå University, SE-901 85 Umeå, Sweden

## Abstract

**Background:**

The prerequisite for the potential use of the bacterial toxin verotoxin-1 in the treatment of breast cancer was investigated by first determining the expression of its receptor Gb3 (CD77) in clinical breast cancer tissue specimens. We then examined the cytotoxicity and mechanism of apoptosis induction of Escherichia coli verotoxin-1 (VT-1) in two human breast cancer cell lines.

**Methods:**

Immunohistochemistry for Gb3 expression was performed on cryostat section from 25 breast cancer specimens. The human breast cancer cell lines T47D and MCF-7 were screened for Gb3 expression by flow cytometry. Fluorescein diacetate and LDH release was used to determine cell viability after VT-1 exposure. Apoptosis was studied by measuring caspase activity and DNA-fragmentation. Signal transduction studies were performed on T47D cells with immunoblotting.

**Results:**

Gb3 expression was detected in the vascular endothelial cells of all tumours specimens, and in tumour cells in 17 of the specimens. We found no associations between tumour cell Gb3-expression and age, tumour size, TNM-classification, histological type, hormone receptor expression, or survival time. T47D cells strongly expressed Gb3 and were sensitive to the cytotoxicity, caspase activation and DNA fragmentation by VT-1, whereas MCF-7 cells with faint Gb3-expression were insensitive to VT-1. VT-1 (0.01 – 5 μg/L) exposure for 72 h resulted in a small percentage of viable T47D cells whereas the cytotoxicity of cells pre-treated with 2 μmol/L D, L-treo-1-phenyl-2-palmitoylamino-3-morpholino-1-propanol (PPMP, an inhibitor of glucosylceramide synthesis) was eliminated (≤ 0.1 μg/L VT-1) or reduced (0.5 – 5 μg/L VT-1). VT-1 did not cause cellular LDH-release or cell cycle arrest. VT-1 induction of caspase-3 (0.1, 1, and 5 μg/L VT-1), -8, and -9 (1 and 5 μg/L VT-1) activity and DNA fragmentation of T47D cells was blocked by PPMP. Key components of MAP kinase signalling pathways that control mitochondrial function were investigated. VT-1 0.1 – 5 μg/L induced phosphorylation of JNK as well as MKK3/6 suggesting that survival signal pathways were overruled by VT-1-induced JNK activation leading to mitochondrial depolarization, caspase-9 activation and apoptosis.

**Conclusion:**

The high specificity and apoptosis-inducing properties of verotoxin-1 indicates that the toxin potentially may be used for treatment of Gb3-expressing breast cancer.

## Background

Breast cancer is the most common malignancy affecting women in the Western world. Systemic chemotherapy is indicated for women with metastatic breast cancer (MBC) [1]. Several single-agent and combination chemotherapeutic options have been shown to be effective as first- or second-line therapy in the management of MBC with the taxanes and anthracyclines being the most active drugs [2], however often with substantial side effects. An important area of cancer research is the search for new effective agents for targeted treatment with as small side effects as possible. Apoptosis, the well-regulated intrinsic suicide program that enables removal of unwanted cells is known to be disabled in many tumour cells and identifying agents that can overcome resistance to apoptosis could greatly improve current chemotherapy options [3,4].

Bacterial toxins such as AC-toxin, botulinum toxin, cholera toxin, and verotoxin have been suggested to be used as an approach to establish novel therapeutic agents against tumour malignancies either as independent anti-neoplastic agents or in combination treatment with chemo- or radiotherapy. Toxins effective against specific signalling pathways could reduce treatment side-effects to normal tissues and be an approach to generate specific anti-tumour agents [5]. VT-1 is a member of the bacterial shigatoxin family expressed by some bacterial serotypes of *Escherichia coli*, and *Shigella dysenteriae*. Verotoxin-1 induces cytotoxicity, apoptosis, and cell cycle arrest in human tumour cells and has been suggested to be an anti-cancer agent candidate due to its low general toxicity and high specificity against tumours expressing its receptor, globotriasosylceramide (Gb3) [6].

Gb3 (CD77) has been reported to be increased on the surface of several tumour cells lines originating from breast cancer, ovarian cancer, haematological malignancies, and astrocytoma tumours as well as normal epithelial cells [7,8]. The targeting of the toxin to a specific intracellular transport pathway is determined by the Gb3 isoform expressed on the cell surface and by the presence or absence of Gb3 in the lipid raft microdomains of the cell membrane [9].

The purpose of this study was to investigate the potential of verotoxin-1 as a novel agent for enhancing apoptosis in breast cancer cells by examining the expression of the verotoxin-1 receptor Gb3 in breast cancer tissue followed by elucidation of the specific signalling pathways to cellular proliferation and apoptosis. We investigated the cytotoxicity and induction of apoptosis as well as the signal transduction mechanisms to apoptosis of *Escherichia coli *verotoxin-1 in human breast cancer cell lines. We demonstrate that verotoxin-1 has the potential to be an effective anticancer drug in Gb3-expressing breast cancer.

## Methods

### Breast cancer patients

Tumour specimens were collected in the northern healthcare region of Sweden between 1985 and 1989 from 25 unselected women with primary invasive breast carcinoma (11 node-negative and 14 node-positive patients) all with snap-frozen tumour tissue available. Node-negative patients were generally treated with a modified radical mastectomy or conservative surgery, followed by radiotherapy. All node-positive patients were treated with surgery and radiotherapy. Adjuvant systemic treatment was administered to all node-positive patients. Most postmenopausal patients also received adjuvant endocrine therapy. Prognostic and biological information were available for all patients regarding TNM-classification, histological type, oestrogen receptor (ER), progesterone receptor (PgR), and survival time. The median age was 64 years (range 34–83 years) and the median follow-up time was 63 months (last follow-up date was 30 June 1998). Samples was collected before informed consent became mandatory, the study was approved (Dnr 02-455) by the ethics committee of Umeå University.

### Gb3 immunohistochemistry

Cryostat sections from biopsies of the breast cancer tumours were cut and post-fixed in acetone. Gb3 immunohistochemistry was performed with a monoclonal rat IgM antibody at a dilution of 1:40 using the Envision system (Dako, Denmark) and the staining evaluated by an experienced pathologist (TB).

### Cell lines and cell culture conditions

Two human breast cancer cell lines T47D and MCF-7 were used. The cells were maintained under standard cell culture conditions, grown as monolayer culture in Eagle's MEM with Earl's salt supplemented by 10% foetal bovine serum and 200 μmol/L L-glutamine. They were incubated at 37°C in a humidified atmosphere containing 5% CO_2_.

### Determination and inhibition of Gb3-expression of cultured cells

T47D and MCF-7 cells were screened for expression of Gb3 (CD77), the receptor for VT-1. Cellular expression of Gb3 was identified by a monoclonal rat IgM antibody on a FACSCalibur flow cytometer (Becton Dickinson Immunocytometry Systems, San Jose, CA). DL-*threo*-1-phenyl-2-palmitoylamino- 3-morpholino-1-propanol (PPMP) a chemical inhibitor of glucosylceramide synthesis was used to quench Gb3 expression. Cells were cultured with 2 μmol/L PPMP for 72 h prior to Gb3 expression analysis.

### Cell viability assays

A fluorometric method utilising fluorescein diacetate (FDA) was used to quantify cell viability and determine VT-1 sensitivity of T47D and MCF-7 cells *in vitro *[10]. Cells (1 × 10^4^) were cultured and incubated with VT-1 (0.1 – 5 μg/L) followed by fluorescence determination as described earlier [11]. The same procedure was made with cells pre-treated with 2 μmol/L PPMP for 72 h prior to VT-1 exposure, in which case 2 μmol/L PPMP was present in the medium during the whole VT-1 incubation.

A colorimetric assay (Cytotoxicity detection kit, LDH Roche Applied Science, Mannheim, Germany) was used as described earlier [12] to measure lactate dehydrogenase (LDH) release following cell membrane damages not seen in apoptotic cells [13] to quantify necrosis induction by VT-1 (0.1 – 5 μg/L) after 6 h.

### Cell cycle analysis with propidium iodide staining measured by flow cytometry

Propidium iodide (PI) is a fluorescence dye that binds specifically to double-stranded nucleic acids [14,15]. In the flow cytometry assay employed, PI fluorescence is indicative of the DNA content of the cells. Cells in the G2/M phase are preparing to divide and they contain double amount of DNA (4*n*) compared to cells in the G1 phase that have not yet replicated their DNA (2*n *DNA content). Upon completion of 72 h incubation with/without 0.01, 0.1 or 1 μg/L VT-1, the cell cultures were washed with PBS and treated with 0.1% trypsin at 37°C. The cell suspension was collected, washed once with PBS (200 g, 5 min), and re-suspended in PBS containing 70% cold absolute ethanol, for fixation and permeabilisation of the cell membrane (1 h, 4°C). After fixation the cells were washed twice with PBS (900 g, 5 min), and the cells were treated with 40 μg/mL RNase in PBS (final volume 100 μL), for 15 min at 37°C. Finally, the PI staining solution (50 μg/mL PI and 3.8 mmol/L sodium citrate in PBS) was added to the cells, followed by 3 h incubation in the dark, at 4°C. The cell cycle analysis was performed by a Fluorescence Activated Cell Sorter (FACSCalibur, Becton Dickinson, San Jose CA USA), and PI fluorescence (designated as FL-2 Height in the histogram plots) was measured at 488 nm. Ten thousand cells were analyzed in each experiment. The percentage of cells in the G2/M phase of the cell cycle was then determined. The same procedure was used on cells pre-treated with 2 μmol/L PPMP for 72 h before VT-1 treatment.

### DNA fragmentation analysis

TUNEL (TdT-mediated dUTP nick end labelling) staining detecting nuclear DNA fragmentation was used as a marker for late stage apoptosis using Roche's In situ cell death detection kit, TMR red (Roche Applied Science, Mannheim, Germany). T47D and MCF-7 cells were cultured to about 80% confluence. Medium was thereafter changed to fresh medium containing 0–5 μg/L VT-1 and incubation continued for 72 h. Cells were thereafter harvested with trypsin and any floating cells collected by centrifugation. Followed by TUNEL-stained according to the manufactures instructions and TUNEL-staining was determined with flow cytometry. The same procedure was used on cells pre-treated with 2 μmol/L PPMP for 72 h before VT-1 treatment.

### Caspase activity determination

Fluorometric assays were used to detect caspase -3, -8, and -9 enzyme activities. T47D and MCF-7 cells were treated with 0–5 μg/L VT-1 for 24 h. Thereafter caspase-enzyme activity was measured with fluorometric assays (R&D Systems Inc., Minneapolis, MN, USA) as earlier described [12]. The same procedure was used on cells pre-treated with 2 μmol/L PPMP for 24 h before VT-1 treatment.

### SDS-PAGE gel electrophoresis and immunoblotting

VT-1 influence on specific proteins involved in apoptosis signal transduction was investigated through Western blotting. Cells were exposed to 0.1, 1, 5 μg/L VT-1, and 5 μg/L VT-1 with 2 μmol/L PPMP for 24 h and then lysed in lysis buffer. Gel electrophoresis and immunoblotting was preformed as earlier described [11] using primary antibodies against AKT, p-Akt_ser473_, p-Akt_tyr308_, Bad, p-Bad_ser112_, p-Bad_ser136_, Bax, Bcl-2, p-Bcl-2_ser70_, Bcl-X_L_, SAPK/JNK, p-SAPK/JNK, P44/42, p-P44/42 (ERK1/2), MKK 3/6, p-MKK 3/6 and Monoclonal β-Actin antibody for detection of actin as loading control.

### Reagents

Antibodies were obtained from the following: rat monoclonal IgM for Gb3 expression were from Immunotech, Marseille, France and all other antibodies were from Cell Signalling Technology Inc. Danvers, MA, USA. Eagle's MEM in Earl's salt was from Gibco Ltd, Paisley, Scotland, UK, and foetal bovine serum and L-glutamine were from Biochrom KG, Berlin, Germany. Trypsin. DL-*threo*-1-phenyl-2-palmitoylamino- 3-morpholino-1-propanol (PPMP), fluorescein diacetate (FDA), verotoxin-1, propidium iodide (PI), and RNase were from Sigma-Aldrich, St. Louis, MO, USA. Lysis buffer were from R&D Systems Inc. Minneapolis, MN, USA. The bicinchoninic acid (BCA) protein assay kit was from Pierce Biotechnology Inc. Rockford, IL, USA. NuPAGE antioxidant and reducing agent were from Invitrogen, Carlsbad, CA, USA. Tris-HCl SDS-PAGE criterion precast gel and Immune-Blot PVDF membranes were from Bio-Rad, Hercules, CA, USA. ECL Advance Western blotting detection system were from Amersham Biosciences, Buckinghamshire, UK.

### Statistics

Statistical significance was tested with one-way ANOVA. The level of significance for rejecting the null hypothesis of zero treatment effect was taken to be p = 0.05.

## Results

### Immunohistochemical expression of Gb3 in breast cancer surgical specimens and patient outcome

**Figure 1:**
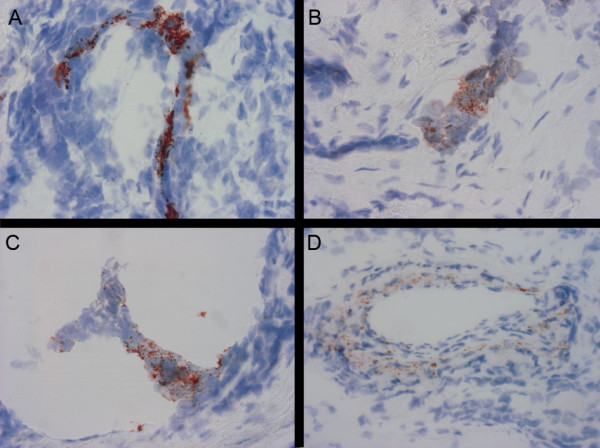
**Micrographs of immunohistochemistry on two breast cancer cryostat sections showing immunoreactivity of Gb3 (redbrown), in (A-C) tumour cells and (D) vascular vessels**. 40× magnification.

Immunohistochemistry of cryostat sections of 25 breast cancers demonstrated Gb3 expression in some arterioles and small arteries in all specimens, whereas Gb3 expression was found in some tumour cells of 17 breast cancer specimens. The vascular Gb3 staining was granular and mainly confined to the endothelium but also seen in the media of larger arterioles and small arteries. Fig. 1A–C illustrates the immunohistochemical staining of two ductal type breast cancer specimen with Gb3 expression in tumour cell expression and endothelial staining in a small arteriole. Fig 1D illustrates the granular Gb3 staining found in the vessel wall of a small artery. There were no apparent associations between tumour cell expression of Gb3 and lymph-node status, tumour size, TNM-classification, histological type or hormone receptor expression at the time of diagnosis (Table 1) nor death of disease, or survival time (not shown). A total of 16 deaths were recorded during the follow-up time.

**Table 1:** Clinicopathological characteristics of the patients

Feature	With Gb3-expressing tumor cells (total number)
**Patients**	17 (25)
	
**Lymph-node status**	
Node-negative	7 (11)
Node-positive	10 (14)
	
**Histological type**	
Ductal	12 (16)
Lobular	1 (2)
Papillary	1 (2)
Medullary	1 (1)
Mixed	2 (4)
	
**Tumor size**	
T1	5 (8)
T2	10 (13)

Clinicopathological characteristics of 25 breast cancer patients

### Gb3 expression of cultured breast cancer cells after verotoxin-1 exposure with/without PPMP inhibition

**Figure 2:**
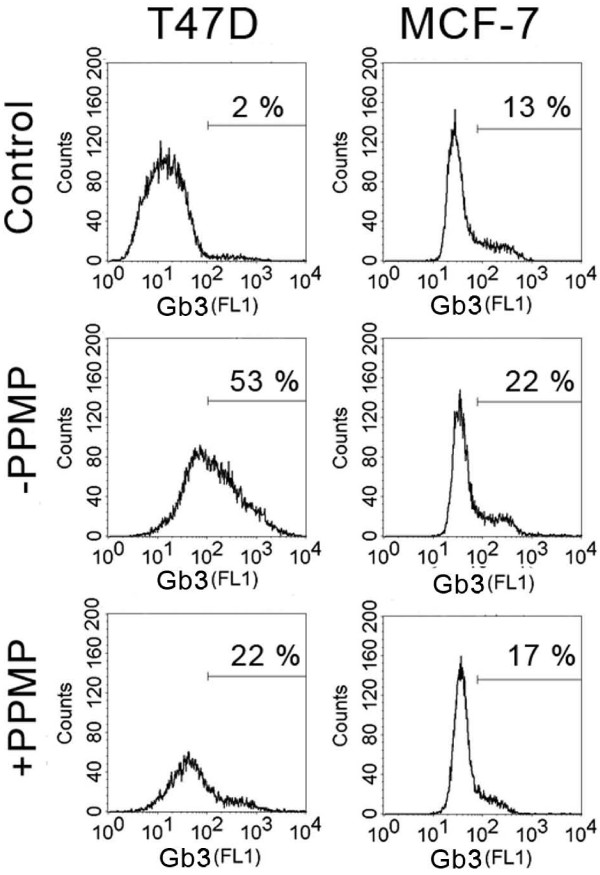
**Gb3 expression of T47D and MCF-7 cells with/without 72 h pre-treatment with 2 μmol/L PPMP**. Control is with secondary antibody only.

We then determined the expression of Gb3 in the human breast cancer cell lines T47D and MCF-7. High Gb3 expression was observed on the surface of T47D whereas Gb3 was only weakly expressed by MCF-7 cells (Fig. 2). Culturing of T47D cells in the presence of 2 μmol/L PPMP for 72 h, lead to a marked inhibition of Gb3 expression.

### Verotoxin-1 cytotoxicity and cell cycle arrest

**Figure 3:**
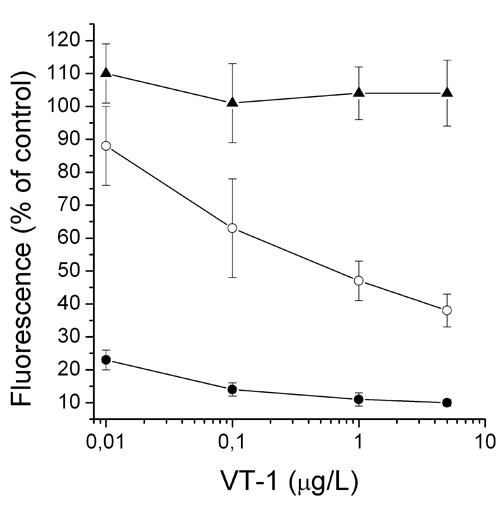
**Viability dose-response of T47D cells (filled circles), PPMP-pre-treated T47D cells (open circles), and MCF-1 cells (triangles), after 72 h of exposure to verotoxin-1 at indicated concentrations 100% cell viability is untreated cells cultured under for 72 h**. Data denote mean values ± SD for 6 or 12 separate observations.

When incubated with 0.01 – 5 μg/L VT-1 for 72 h, the number of viable T47D cells with high expression of Gb3 were very sensitive to VT-1 and the number of surviving cells low even at 0.01 μg/L VT-1 (Fig. 3). When pre-incubated with PPMP for 72 h, full protection against VT-1 cytotoxicity was noted at 0.01 μg/L VT-1 whereas PPMP protection at 0.1, 1 and 5 μg/L VT-1 was reduced with increasing toxin concentration. On the other hand, VT-1 failed to induce cytotoxicity on the MCF-7 cell line with low expression of Gb3 at a concentration as high as 5 μg/L VT-1 (Fig. 3). Involvement of cytotoxicity by cell membrane disruption was investigated by analyzes of LDH-release after 6 h of VT-1 treatment. VT-1 (up to 5 μg/L) did not increase LDH-release in T47D cells (data not shown). Cell cycle analysis with propidium iodide staining for 24, 48 or 72 h demonstrated no effect of VT-1 on the distribution of cells in different cell cycle phases compared to untreated control (data not shown).

### Verotoxin-1 induction of DNA-fragmentation

**Figure 4:**
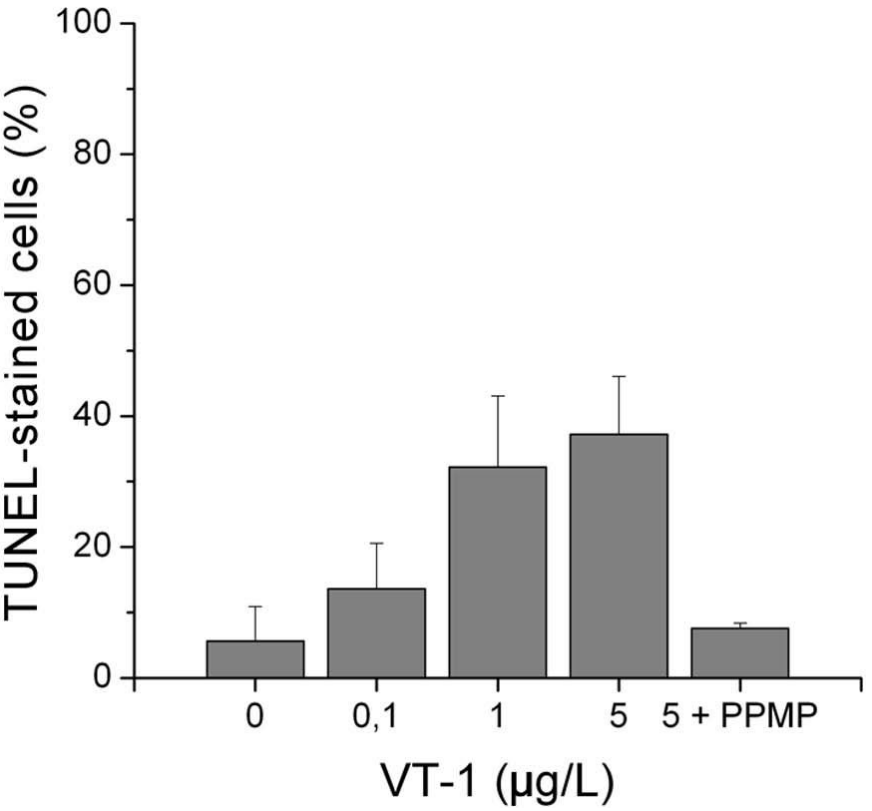
**Percentage of TUNEL-stained T47D cells after 72 h exposure to 0, 0.1, 1, or 5 μg/L verotoxin-1 and μg/L verotoxin-1 with 2 μmol/L PPMP Mean ± SD of three separate observations**.

DNA-fragmentation as marked by TUNEL-staining increased in T47D cells when they were incubated with 0, 0.1, 1, or 5 μg/L VT-1 for 72 h. Pre-incubation with 2 μmol/L PPMP for 72 h completely inhibited TUNEL-staining of T47D cells induced by incubation with 5 μg/L VT-1 (Fig. 4).

### VT-1 induction of caspase activity in T47D cells

**Figure 5:**
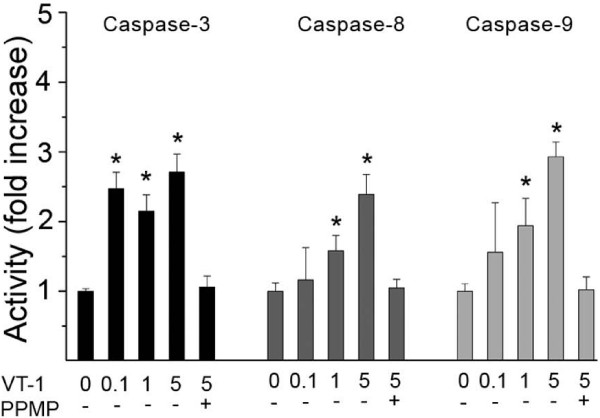
**Relative caspase-3, -8 and -9 enzyme activities of T47D cells incubated for 24 h with verotoxin-1 with or without 2 μmol/L PPMP compared to untreated control (activity set to 1)**. Significant differences (p < 0.05) compared to untreated control are marked with *.

To elucidate the signal transduction pathways of VT-1-induced apoptosis, the induction of caspase-3, -8, and -9 enzyme activity was examined. Caspase-3 enzyme activity increased when T47D cells were incubated with 0.1, 1 and 5 μg/L VT-1, and caspase-8, and -9 enzyme activity increased when the were incubated with 1 and 5 μg/L VT-1 for 24 h. When pre-incubated with 2 μmol/L PPMP for 24 h, all caspase enzyme activity was eradicated even with cells incubated with 5 μg/L VT-1 (Fig. 5).

### Immunoblot analysis

**Figure 6:**
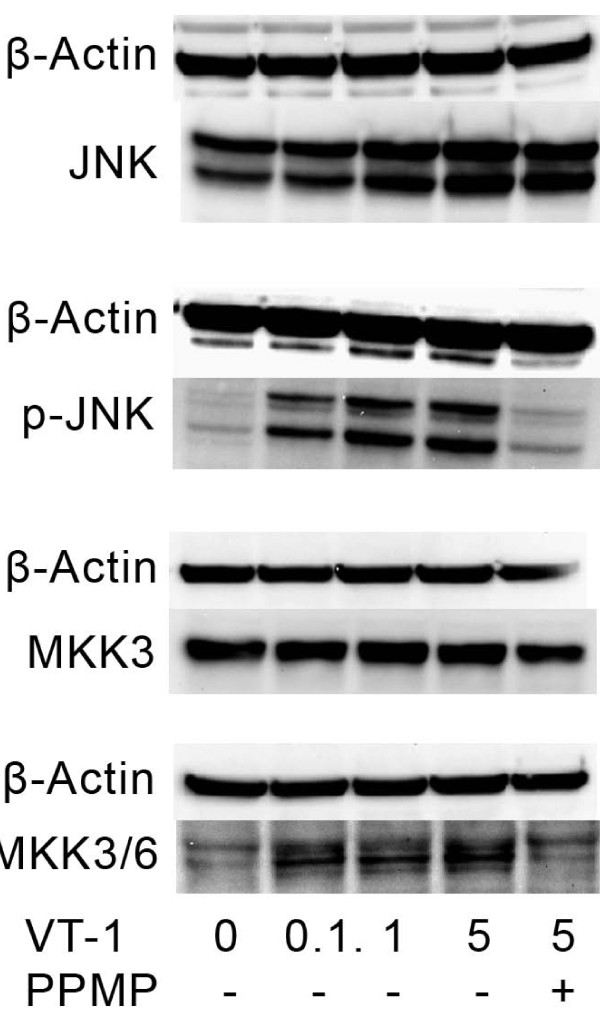
**Effect of verotoxin-1 in T47D cells on the expression of the total and phosphorylated MAP kinases JNK and MKK3/6 after 24 h exposure to verotoxin-1 with or without 2 μmol/L PPMP**. β-Actin is shown as loading control.

Western blot analysis was performed in an attempt to identify involvement of pro- and anti-apoptotic proteins (Akt, p-Akt_ser473_, p-Akt_tyr308_, Bad, p-Bad_ser112_, p-Bad_ser136_, Bax, BCL-2, p-Bcl-2_ser70_, Bcl-X_L_, SAPK/JNK, p-SAPK/JNK, P44/42, p-P44/42 (ERK1/2), MKK 3/6, p-MKK 3/6) upstream of caspase-3. T47D cells were incubated with 0, 0.1, 1, 5 μg/L of VT-1, and 5 μg/L VT-1 with 2 μmol/L PPMP for 24 h. SAPK/JNK (stress activated protein kinase/c-JUN N-terminal Kinase), Bad, MKK3/6, and Akt were uniformly expressed with no expression differences regardless of treatment. The similar consistent pattern of expression was noted for phosphorylated Akt at threonine 308 and serine 473. The active form of SAPK/JNK (p-SAPK/JNK) was expressed at phosphorylation-sites threonine 183 and tyrosine 185 in cells incubated with 0.1, 1, and 5 μg/L of VT-1 but not in control cells, nor in cells pre-treated with PPMP for 72 h (Fig. 6). The same expression pattern was detected for phosphorylated MKK 3 and 6, at serine 189 and threonine 193 for MKK3, and serine 207 and threonine 211 for MKK6. Phosphorylation of Bad at serine 112 and 136 was constitutively expressed regardless of VT-1 concentrations. Bax, Bcl-2, Bcl-X_L_, ERK1 and 2 (p44/42 MAP kinase) showed no changes from VT-1 treatment (data not shown).

## Discussion

The use of toxins targeted against specific tumour cells or signal transduction pathways could reduce cancer treatment side effects and toxicity to normal tissues and thus be an approach to generate specific antitumour effects [16]. Our results demonstrate that verotoxin-1- (VT-1) induced cytotoxicity involves induction of apoptosis in breast cancer cells expressing the toxin receptor Gb3. Gb3 expressing T47D cells were highly sensitive to VT-1 *in vitro*, In contrast, the MCF-7 cells lacking Gb3 were completely resistant to VT-1 treatment. Moreover, using PPMP, that inhibits glucosylceramide synthesis and thus makes exposed cells unable to synthesize Gb3 [17] rendered T47D cells resistant or partly resistant to VT-1 depending on toxin concentration.

We also demonstrated that VT-1 at higher concentrations induces the intrinsic pathway to apoptosis, probably through activation of JNK. This leads to disruption of the mitochondrial membrane potential, activation of caspase-9 and -3, ultimately leading to DNA fragmentation and cell death. At higher VT-1 concentrations the mode of cell death was still through Gb3-dependent apoptosis. This was indicated by complete PPMP-blockable VT-1-induced MAP kinase protein expression, caspase activities and DNA fragmentation.

Most chemotherapeutic drugs exert their action by inducing apoptosis and the development of resistance to chemotherapy appears to be mediated by alterations in the sensitivity towards apoptosis [18,19]. Progress of tumour growth and resistance to treatment is associated with decreased rate of apoptosis [20]. Many cytotoxic agents have a relatively narrow therapeutic window and in many patients the risk of side effects are higher than the probability of obtaining a clinical benefit. Targeted toxins against specific pathways could reduce the toxicity to non-tumour cells and be an approach to generate specific anti-tumour agents.

Several reports have demonstrated that VT-1 induces apoptosis in solid tumour cell lines such as astrocytoma, renal cell carcinoma, and colon cancer [21-23]. VT-1 has also been shown to induce apoptosis and rapid elimination of mice tumour xenografts in Gb3-expressing human renal cell carcinoma, colon carcinoma, bladder carcinoma, glioblastoma, malignant meningiomas [23-27].

Furthermore, 80% of all primary and metastatic breast cancer biopsies have been shown to express Gb3 [8]. A recent study demonstrated expression of Gb3 in metastatic colon cancer, whereas expression was virtually absent in normal colonic epithelial cells [26]. Elevated expression of Gb3 has also been demonstrated in drug-resistant ovarian tumours compared to the primary tumour [28]. Gb3 has also shown to co-localize with the multi drug resistance protein P-glycoprotein (Pgp/MDR1) and have thus been suggested as a possible target for multi drug resistant cells [29]. We recently demonstrated Gb3 in vascular endothelial- and tumour cells by immunostaining of Gb3 expression in glioma cryostat sections. The present results of the immunostaining showed expression of Gb3 in endothelial cells but also in tumour cells. This suggests that VT-1 exposure could be effective directly against breast cancer tumour cells but also affect tumour vascularity as suggested also for gliomas [30]. The present demonstration of Gb3 expression in breast cancer tissue indicates that it is possible to use VT-1 also in the treatment of breast cancer. Further investigations should, however, be conducted to ensure that the cytotoxic effect of VT-1 does not lead to severe adverse effects due to targeting of normal Gb3-expressing cells outside of the tumor. The most important target expressing high levels of Gb3 would be the microvasculature of the kidney. It is also the main target of E. Coli VT-1-induced haemolytic uraemic syndrome [31] Other non-tumour target cells of concern for adverse effects would be tonsil germinal-center B lymphocytes which regularly express Gb3 [32]. Finding safe modes of administration are of critical importance for use of VT-1 as an anti-neoplastic agent.

We demonstrated that VT-1 induced a dose-dependent decrease of cell viability in Gb3-expressing T47D cells, but not in MCF-7 cells which lacked Gb3 expression. Expression of the membrane receptor Gb3 is obviously required for VT-1 internalization and transport of the toxin to the endoplasmic reticulum [33]. When T47D cells were incubated with PPMP, that inhibits glucosylceramide synthesis and thus Gb3 expression [17], prior to VT-1 exposure the sensitivity to VT-1 cytotoxicity at lower toxin concentrations was lost.

We then studied if VT-1-induced cytotoxicity was due to induction of apoptosis. Massive VT-1-induced DNA-fragmentation as well as apoptosis-related morphological changes was detected in the Gb3-expressing cell line T47D after 72 h incubation with high concentrations of VT-1. Pre-treatment with PPMP for 72 h prior to VT-1 exposure completely abolished VT-1-incuded DNA fragmentation. Apoptosis is most often executed by the activation of caspase -3. Activation of caspase-3 can be achieved either by caspase-8, the most proximal caspase in the receptor signalling pathway or by caspase-9 in the mitochondrial pathway to apoptosis. We therefore examined the activation of the three caspases after 24 h VT-1 exposure. Caspases-3 and -9, but not caspase-8 was found to be activated in both cell lines indicating that the intrinsic pathway to apoptosis was activated by VT-1. There was no VT-1-induced cellular activation of caspase-3,-8, or -9 after pre-incubation with PPMP. The mode of VT-1 cytotoxicity at low concentrations is still unclear. Nevertheless, cytotoxicity is apparently dependent on the cellular expression of Gb3 but not due to apoptosis, necrosis or cell cycle arrest as noted by lack of caspase activity or DNA fragmentation, cell lysis, and changes in cell cycle distribution, respectively.

Protein phosphorylation is the major cellular mechanism used to regulate protein functions, among them controlling cell growth, death, differentiation and apoptosis. The phosphorylation state of the key components of MAPK signalling pathways [34] controlling Bax and mitochondrial function related to cell survival and apoptosis was therefore investigated. VT-1 induced JNK and MKK3/6 phosphorylation in T47D cells, suggesting that survival signal pathways were overruled by VT-1-induced JNK and p38 activation leading to mitochondrial depolarization, caspase-9 activation and apoptosis. Similar increases of JNK phosphorylation leading to apoptosis have been demonstrated to be essential for *Pseudomonas aeruginosa *ExoS-induced apoptosis [35] and for verotoxin-1 in glioma cell lines [11].

Despite clinical improvements there are still major problems associated with breast cancer treatment. Chemotherapy and radiotherapy are associated with many, sometimes severe side effects. Therefore, targeted therapies that effectively kill cancer cells, without affecting normal tissues is a major objective in clinical cancer research. The high specificity and the ability of verotoxin-1 to selectively induce breast cancer cell death indicate that verotoxin-1 may be used as a potential anti-neoplastic agent for treatment of Gb3-positive breast cancers.

## Conclusion

Despite clinical improvements there are still major problems associated with breast cancer treatment. Chemotherapy and radiotherapy are associated with many, sometimes severe side effects. Therefore, targeted therapies that effectively kill cancer cells, without affecting normal tissues is a major objective in clinical cancer research. The high specificity and the ability of verotoxin-1 to selectively induce breast cancer cell death indicate that verotoxin-1 may be used as a potential anti-neoplastic agent for treatment of Gb3-positive breast cancers.

## Competing interests

The authors declare that they have no competing interests.

## Authors' contributions

DJ, PBM and AJ conceived and designed the study. EK and JM carried out most of the assays. IL collected the clinical data and TB evaluated the immunohistochemical staining. The manuscript was prepared by DJ, PBM and AJ. All authors read, critically advised, and approved the manuscript.

## Pre-publication history

The pre-publication history for this paper can be accessed here:

http://www.biomedcentral.com/1471-2407/9/67/prepub
